# Socio-demographic determinants of dietary choices and their impact on health in Spanish adults

**DOI:** 10.3389/fpubh.2024.1417925

**Published:** 2024-11-07

**Authors:** Elena Sandri, Eva Cantín Larumbe, Michela Capoferri, Germán Cerdá Olmedo, Lisa Ursula Werner, M. Jesús Vega-Bello

**Affiliations:** ^1^Faculty of Medicine and Health Sciences, Catholic University of Valencia San Vicente Mártir, Valencia, Spain; ^2^Doctoral School, Catholic University of Valencia San Vicente Mártir, Valencia, Spain; ^3^Degree in Data Science, Polytechnical University of Valencia, Valencia, Spain; ^4^Faculty of Teaching and Science of Education, Catholic University of Valencia San Vicente Mártir, Valencia, Spain

**Keywords:** diet food and nutrition, healthy lifestyle, survey, Spain, socioeconomic factors

## Abstract

**Background:**

Although Spain has traditionally followed the Mediterranean diet, in recent years, changes have become noticeable in the food preferences of the population. These changes include adopting new diets and dietary trends, such as plant-based diets, intermittent fasting and raw food diets. These choices are influenced by cultural, social and socio-demographic factors, and their impact on health should be studied in detail.

**Aim:**

The objectives of this research are: (1) to study the prevalence of different dietary patterns among the Spanish population, (2) to explore the dependence of dietary choice on socio-demographic factors, (3) to investigate the relationship between the social habits and lifestyle choices of the population and how it affects health.

**Methods:**

A descriptive cross-sectional study was carried out on the Spanish population. Using a questionnaire constructed and validated by the research team, socio-demographic data were collected and different nutritional, social and lifestyle habits of the targeted population were explored.

**Results:**

A valid sample of 22,181 people was collected among which 17,573 (79.2%) people claimed to follow a Mediterranean dietary pattern, 1,425 (6.4%) people followed a plant-based diet, 365 (1.6%) people are vegans, 1,018 people (4.6%) practiced intermittent fasting, 252 (1.1%) people followed a raw food diet and 1,548 people (7%) claimed to follow other types of diets. The data show that younger people (18–25 years old) tend to adopt more often a plant-based diet than older people and that women tend to follow this type of diet more often than men. On the other hand, men seem to practice intermittent fasting more frequently. A higher tendency to practice a raw food diet was found among people living in villages than those living in cities. Moreover, some trends were found in different regions of Spain, with greater adherence to a plant-based diet in Catalonia, while intermittent fasting is more common in the Region of Murcia. Finally, the results indicate that individuals who pay attention to their diet and experiment with various dietary patterns are generally more health-conscious. This is reflected in their adoption of healthier behaviors, such as exercising more and reducing their intake of alcohol and sugary drinks.

## 1 Introduction

The alimentary patterns adapted by the people play a crucial role in the overall health and wellbeing of a population (1, 2). Understanding food-related behaviors, physical activity patterns and other lifestyle factors is of great importance in nutritional epidemiology research and can lead to significant changes in planning public health initiatives, dietary guidelines, and health interventions (3).

In Spain, the traditionally followed Mediterranean diet (MD) has long been recognized for its health benefits (4). It is characterized by a high intake of fruits and vegetables, olive oil as the primary fat source, reduced meat and dairy consumption, and moderate red wine intake. The Mediterranean diet also includes eating fish and legumes two to three times a week. These dietary habits contribute to the reduction of cardiovascular disease risks such as hypertension, hypercholesterolemia, and obesity (5, 6). Although the MD is well-established, other dietary patterns have gained popularity (7, 8), and their health impacts are currently the subject of various studies (9–17). The most noticeable are the plant-based diets (PBD) (18, 19), which include a range of different diets characterized by reduced animal-based food consumption (20). PBDs have gained popularity for their perceived health benefits (9–11, 17), environmental sustainability (21, 22), and ethical considerations (23).

Within PBDs, the vegetarian diet (VD) and the vegan diet (VG) are the two most known patterns. While the VD excludes meat and, in some cases, some animal-derived products, the VG eliminates all animal-related foods (24). Within the VD group, subgroups can vary in their dietary restrictions, for example, lacto-vegetarians (LV) consume dairy products, and lacto-ovo-vegetarians (LOV) include both eggs and dairy into their food catalog (24). Pesco-vegetarians or pescetarians, who belong to another VD subgroup, include fish, eggs, and dairy but exclude poultry and red meat (25). The raw food diet (RD) emphasizes the consumption of uncooked and unprocessed food. Depending on individual philosophies and goals (26), the RD may include fruits, vegetables, nuts, seeds, eggs, fish, meat, and dairy, often excluding pasteurized or processed foods (27). While vegetarian and vegan diets have been widely studied (9–11, 17), there is limited research on the RD in humans compared to animals (28, 29), highlighting the need for further investigation.

Intermittent calorie restriction (ICR) or intermittent fasting (IF) is another emerging dietary behavior focusing on meal timing rather than specific food choices (30, 31). Continuous calorie restriction (CCR) is commonly recommended for weight loss and managing obesity-related diseases (32), but in the last years, IF is gaining popularity and scientific attention outside of the diet culture, too. IF alternates between periods of food restriction and periods of regular meal intake (33). There are two main types of IF, while in the first form, practitioners alternate between days with restricted food intake and days with normal food intake, like in the 5:2 method, where two out of seven days of the week are used to fast, followers of the second type restrict the time of the day in which food is consumed, like in the 16:8 method, where 8 h a day are used to eat and 16 h remain without food intake (34).

Research on emerging dietary patterns has primarily focused on health impacts (9–17) and the motivations for adopting VD, VG, or IF (35, 36). However, there is a lack of studies connecting the prevalence of these diets and their associations with social, lifestyle, and socio-demographic factors. In Spain, Acevedo Cantero et al. (19) studied the rise of PBDs from 2001 to 2017 and their correlation with physical activity, tobacco and alcohol consumption, and the BMI. That study indicated that individuals who consume alcohol are overweight or obese and are significantly less likely to follow a plant-based diet. These results suggest that certain lifestyle and health-related factors may reduce the likelihood of adopting a plant-based eating pattern, but broader health implications remain understudied.

### 1.1 Study objective

To contribute to the understanding of the impact of emerging dietary patterns on the health and habits of the Spanish population, the present study has three objectives. First, to examine the prevalence of various dietary patterns, namely vegetarian, vegan, intermittent fasting and raw food diets, compared to the Mediterranean dietary pattern. Next, to elucidate whether specific socio-demographic factors, such as age, gender, educational level, income, urbanity or geographic region, influence the adoption of particular diets. And finally, to delineate the association between dietary choices and the social and lifestyle behaviors adopted by individuals within the population and some of their health consequences.

## 2 Methods

### 2.1 Type of study and sampling

A descriptive cross-sectional study was conducted to examine the Spanish population residing in Spain. People under 18 and those with chronic diseases or temporary conditions that could affect their diet were excluded.

### 2.2 Ethical approval

The research adhered to the principles described in the Declaration of Helsinki (37), with emphasis on the ethical treatment of study participants. Approval of the study protocol was obtained from the Research Ethics Committee of the Catholic University of Valencia, with the assigned approval code UCV/2019–2020/152. This ethical oversight ensures that the study was conducted with due regard for the welfare, rights and privacy of the participants. All participants gave their consent to participate in this research.

### 2.3 Instrument

The analyzed data were collected through the online distribution of a self-developed questionnaire (named NutSo-HH: Nutritional and Social Healthy Habits) using snowball probability sampling. Consisting of 53 questions, the questionnaire aimed to collect socio-demographic data on the population and explore different health dimensions. One dimension reported information on the frequency of consumption of various foods and drinks. Another dimension dealt with physical activity habits and levels of sedentary behavior. A further dimension explored social habits such as alcohol and tobacco consumption or frequency of nights out. Moreover, we studied the population’s rest habits, and finally, a last section investigated eating disorders or symptoms of eating disorders in the respondents.

Rigorous methodological approaches were followed for the development of the instrument and psychometric testing and its validity and reliability were supported (38).

### 2.4 Data collection

The primary channel for disseminating the questionnaire was an Instagram account (@elretonutricional) created explicitly for this purpose. Professionals and influencers were contacted through this account to enhance the outreach of the questionnaire. The personal networks of the researchers, including LinkedIn, Twitter, WhatsApp, and Facebook, were also leveraged. The questionnaire was also disseminated to capture those with little or no use of social networks. To this end, e-mails were sent to various associations and establishments throughout Spain, selected for the diversity of their public (pharmacies, clinics, etc.), and they were asked to help with the dissemination. The establishments that agreed to collaborate displayed a poster on their premises explaining the research objectives and containing a QR code that allowed access to the questionnaire to answer it.

The questionnaire remained open for online responses from August 2020 to November 2021. Participants were encouraged to share the questionnaire with their networks, contributing to expanding the study’s reach through referrals and subsequent participation.

### 2.5 Variables

Various socio-demographic variables were captured in this study, encompassing sex, age, region of residence, size of the municipality of residence, employment status, level of education, and income level. Additionally, anthropometric and health-related variables were collected, including weight, height, and self-perceived health status. Another essential dimension of the questionnaire is the variables that explore the respondents’ behaviors to detect the possible presence of eating disorders. These include the variables of concern about gaining weight or embarrassment after eating, lack of control over food intake, concern or dissatisfaction with body image and diagnosed eating disorders. Nutritional habits were a focal point, with data on the number of meals per day and the frequency of consumption of different foods and beverages—this diverse set of variables aimed to provide a holistic understanding of the participants. Regarding the type of diet, the questionnaire had a question in which respondents were asked about the type of diet followed. This answer was closed and allowed only one possible answer, to avoid the occurrence of subjects belonging to more than one dietary category at the same time. Finally, additional health-related habits were documented, including the level of sedentary behavior and physical activity, social habits, sleep patterns, tobacco use, and alcohol consumption characteristics.

#### 2.5.1 Healthy eating index

The IASE (Healthy Eating Index for the Spanish population) was computed using a condensed version of the index, validated by Norte and Ortiz (39). This version incorporates variables such as “fruit,” “vegetables,” “meat,” “dairy,” “cereals,” “pulses,” and “soft drinks.” The index assesses the frequency of consuming foods recommended daily and weekly, as well as those intended for occasional consumption. It also considers dietary variety, a crucial aspect of healthy eating. Each item on the index carries a maximum score of 10, indicating compliance with the recommendations set forth by the Spanish Society for Community Nutrition (SENC) (40). The overall maximum score for the index is 73. This scoring system allows for a quantitative evaluation of adherence to dietary recommendations, providing insights into the participants’ overall dietary patterns in relation to established health guidelines. The categorization of the population’s nutritional habits relies on the derived IASE score, leading to three distinct groups:

“Healthy” (58.4 < IASE ≤ 73). This group reflects behaviors aligning with the recommended nutritional guidelines, suggesting a commendable adherence to a healthy dietary pattern.

“Needs changes” (36.5 ≤ IASE ≤ 58.4). Individuals falling into this category may benefit from dietary adjustments, as their nutritional habits indicate areas where improvements could be made to better align with recommended guidelines.

“Unhealthy” (IASE < 36.5). This group presents nutritional habits that may require significant modifications. Individuals in this category may need interventions and support to transition toward a healthier and more balanced diet.

### 2.6 Categorization of variables

Nutritional variables and health habit variables not addressed by the IASE were categorized on a Likert scale ranging from 1 to 4 points, where a score of 1 means no or low frequency and a score of 4 corresponds to maximum frequency. For the variables of concern about food intake, lack of control or dissatisfaction with body shape, a Likert scale from 1 to 6 was used, where 1 represents “Never” and 6 represents “Always” (Table 1). Finally, body mass index (BMI) and minutes of exercise per week were used as numerical values.

**Table 1:** Categorization of the health and lifestyle variables.

Variable	Category	Score
Sleeping hours	<6 h	1
	6–7 h	2
	7–8 h	3
	>8 h	4
Getting up rested	Never	1
	Very seldom and sometimes	2
	Frequently and almost always	3
	Always	4
Sleep quality	0 and 1	1
	2	2
	3	3
	4 and 5	4
Water	Never and very rarely (2 max. Per month) and 1 glass/cup/week and 2 or more glasses/cups/week	1
	2 glasses/cups or less every day	2
	3–5 glasses every day	3
	More than 5 glasses every day	4
Sugary soft drinks, coffee and energy drinks, Juice	Never and very rarely (2 glasses max. Per month)	4
	One glass per week and 2 or more glasses per week	3
	2 glasses or less every day	2
	3 to 5 glasses and more than 5 glasses every day	1
Consumption of fast food, fried and ultra-processed dishes	Never	1
	Very seldom (2 times a month maximum)	2
	Once a week	3
	Several times a week	4
Getting drunk	Never or less than once a month	1
	Monthly	2
	Weekly	3
	Daily or almost daily	4
Alcohol consumption	Never or once a month	1
	2–4 times a month	2
	2–3 times a week	3
	4–5 times a week or every day	4
Smoking	Non-smoker	1
	Light smoker (less than 5 cigarettes per day)	2
	Moderate smoker (6–15 cigarettes per day)	3
	Severe smoker (more than 16 cigarettes per day)	4
Night outings	Never and sporadically	1
	Between a and 2 night a week	2
	More than 3 times a week	3
	Every day	4
Sedentary lifestyle	<7 h	1
	Between 7 and 9 h	2
	Between 9 and 11 h	3
	More than 11 h	4
Obesophobia, No control, Body image	Always	6
	Very frequently	5
	Frequently	4
	Occasionally	3
	Rarely	2
	Never	1

### 2.7 Data analysis

The collected data were systematized, including the necessary preparation and codification of the different variables, in Excel and later transferred to Jamovi (Version 2.3.28.0) (41) for further analysis. Discrete variables are presented as absolute values and percentages, while continuous variables are expressed as mean and standard deviation.

As a first step, the sample was analyzed for normality using the Shapiro–Wilk test, which, against the standard affirmation that a large size sample is more likely to follow a normal distribution, indicated that none of the variables studied complied with normality. This result could be due to the test’s sensitivity, especially in samples that size. That led us to analyze the data further by plotting them in Q-Q plots, which confirmed the hypothesis of non-normality (42). Thus, we chose the Chi-Square Test for categorical variables and the non-parametric Mann–Whitney *U* test for independent samples for ordinal or numeric variables. Depending on the context, in some cases the diet of interest was established as the control group, and the rest were grouped, in other cases, just two diets were compared while we ignored the other diets. That allowed us to always compare just two different groups, which is a mandatory precondition for the Mann–Whitney *U* test. The significance level was established at 0.05.

Furthermore, we incorporated the effect size (Cramer’s V for the Chi-Square test and the effect size r for the Mann–Whitney *U* test) to determine the size of the differences found in correlation and therefore, their significance. An effect size <0.3 defines a small effect, representing only a minor but noticeable tendency (42). Considering the magnitude of our sample, we classified effect sizes larger than 0.3 as large effects and therefore statistically more significant. All variables were analyzed and compared separately.

## 3 Results

### 3.1 Characteristics of the sample

The socio-demographic characteristics of the sample are presented in Table 2. Most respondents (80.8%) are female, while 19.2% are male. The average age of the participants is 34.9 years. 68.3% of the contestants claimed to have higher education, while 31.7% had basic education. Regarding economics, 43.9% classified their income as low whereas 47.9% receive a medium-high income, and 8.3% didn’t want to disclose this information. Most respondents (79.3%) live in urban areas with a population of over 10,000 habitants.

**Table 2:** Sample and socio-demographic characteristics (*N* = 22,181).

	Mean (SD) or *N* (%)
Male	4,251 (19.20%)
Female	17,930 (80.80%)
Ages (years)	34.9 (11.70)
Male age (years)	36.5 (13.40)
Female AGE (years)	33.0 (11.20)

	Total	Mediterranean diet	Vegetarian	Vegan	Fasting	Raw	Others
Total		17,573 (79.20%)	1,425 (6.40%)	365 (1.60%)	1,018 (4.60%)	252 (1.10%)	1,548 (7.00%)
Age							
18–25	5,952 (26.83%)	4,829 (81.13%)	553 (9.29%)	134 (2.25%)	179 (3.00%)	20 (0.34%)	237 (3.98%)
25–40	9,644 (43.48%)	7,479 (77.55%)	647 (6.70%)	168 (1.70%)	483 (5.00%)	146 (1.53%)	721 (7.48%)
41–65	6,390 (28.81%)	5,096 (79.75%)	222 (3.47%)	63 (0.99%)	352 (5.51%)	86 (1.35%)	571 (8.93%)
> 65	195 (0.88%)	169 (86.67%)	3 (1.54%)	0 (0.00%)	4 (2.05%)	0 (0.00%)	19 (9.74%)
Level education							
Basic education	7,027 (31.68%)	5,528 (78.67%)	443 (6.63%)	127 (1.81%)	294 (4.18%)	69 (0.98%)	566 (8.05%)
Higher education	15,154 (68.32%)	12,045 (79.48%)	982 (6.48%)	238 (1.57%)	724 (4.77%)	183 (1.21%)	982 (6.48%)
Income level							
Low	9,727 (43.85%)	7,549 (77.60%)	703 (7.23%)	204 (2.10%)	446 (4.59%)	197 (2.03%)	718 (7.38%)
Medium- high	10,616 (47.86%)	8,549 (80.53%)	590 (5.56%)	132 (1.23%)	510 (4.80%)	134 (1.26%)	701 (6.60%)
Do not know-no answer	1839 (8.29%)	1,475 (80.21%)	132 (7.18%)	29 (1.58%)	62 (3.37%)	11 (0.60%)	129 (7.01%)
Municipality							
<2000	1,014 (4.57%)	795 (78.40%)	60 (5.92%)	20 (1.97%)	41 (4.04%)	21 (2.07%)	76 (7.50%)
2000–10,000	3,587 (16.17%)	2,800 (78.05%)	229 (6.38%)	56 (1.56%)	169 (4.71%)	51 (1.42%)	282 (7.86%)
>10,000	17,580 (79.26%)	13,977 (79.51%)	1,136 (6.46%)	289 (1.64%)	808 (4.60%)	180 (1.02%)	1,190 (6.77%)

**Figure 1:**
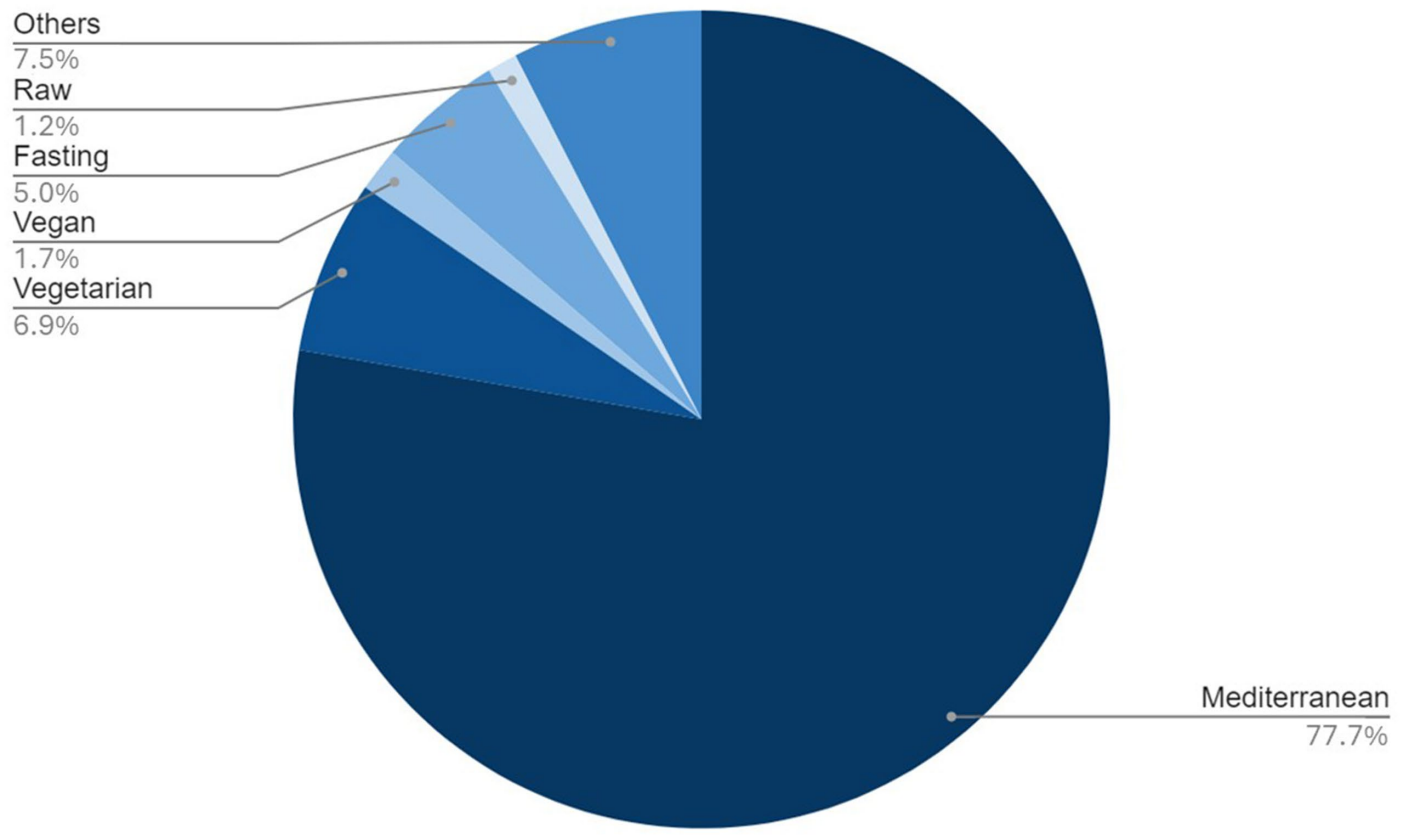
Types of diets followed by the population and their percentages.

Table 2 also describes the socio-demographic dimensions divided by the different dietary choices. Figure 1 shows that, out of the full sample (*N* = 22,181), 79.2% of the contestants follow a Mediterranean diet, 6.4% are vegetarian, 4.6% practice intermittent fasting, 1.6% are vegan, 1.1% eat only unprocessed or raw foods, and 7% follow other diets. The study only focuses on analyzing the Mediterranean, vegetarian, vegan and raw food diets, and the intermittent fasting, the “Other” category was excluded due to the wide range of patterns that would not allow clear conclusions.

### 3.2 Prevalence of the type of diet in the regions of Spain

**Figure 2:**
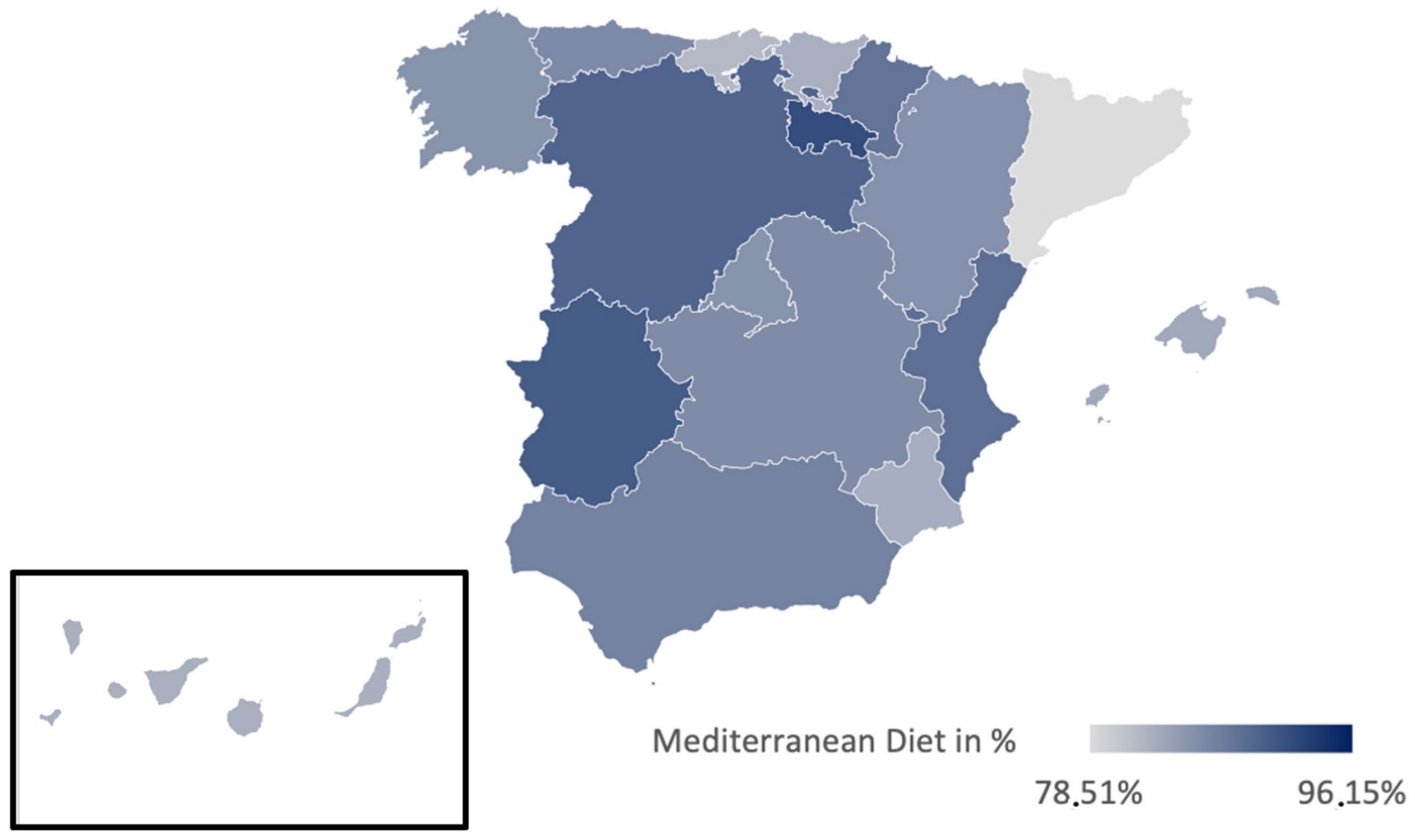
Prevalence of the Mediterranean diet in the different regions of Spain.

**Figure 3:**
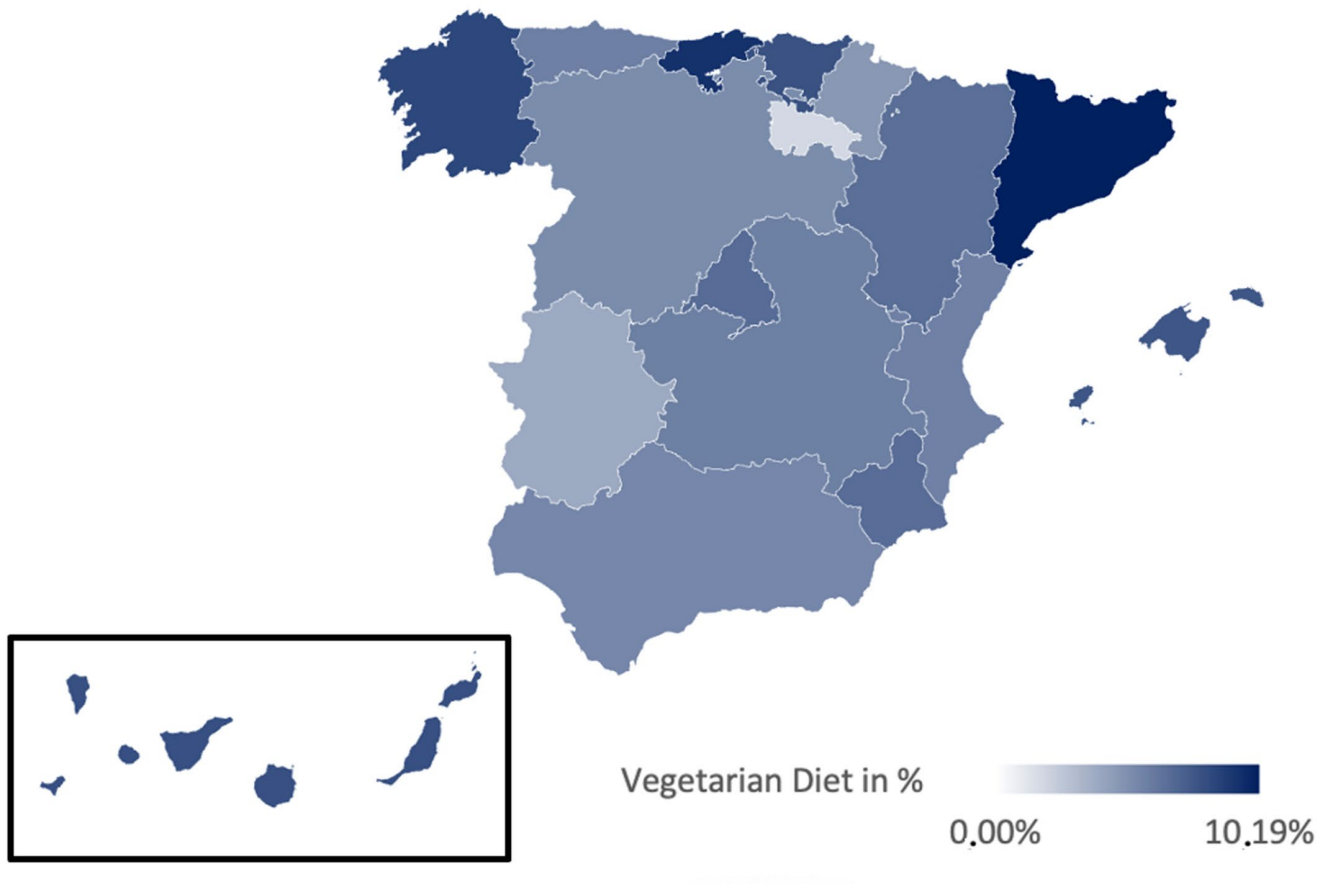
Prevalence of the Vegetarian diet in the different regions of Spain.

**Figure 4:**
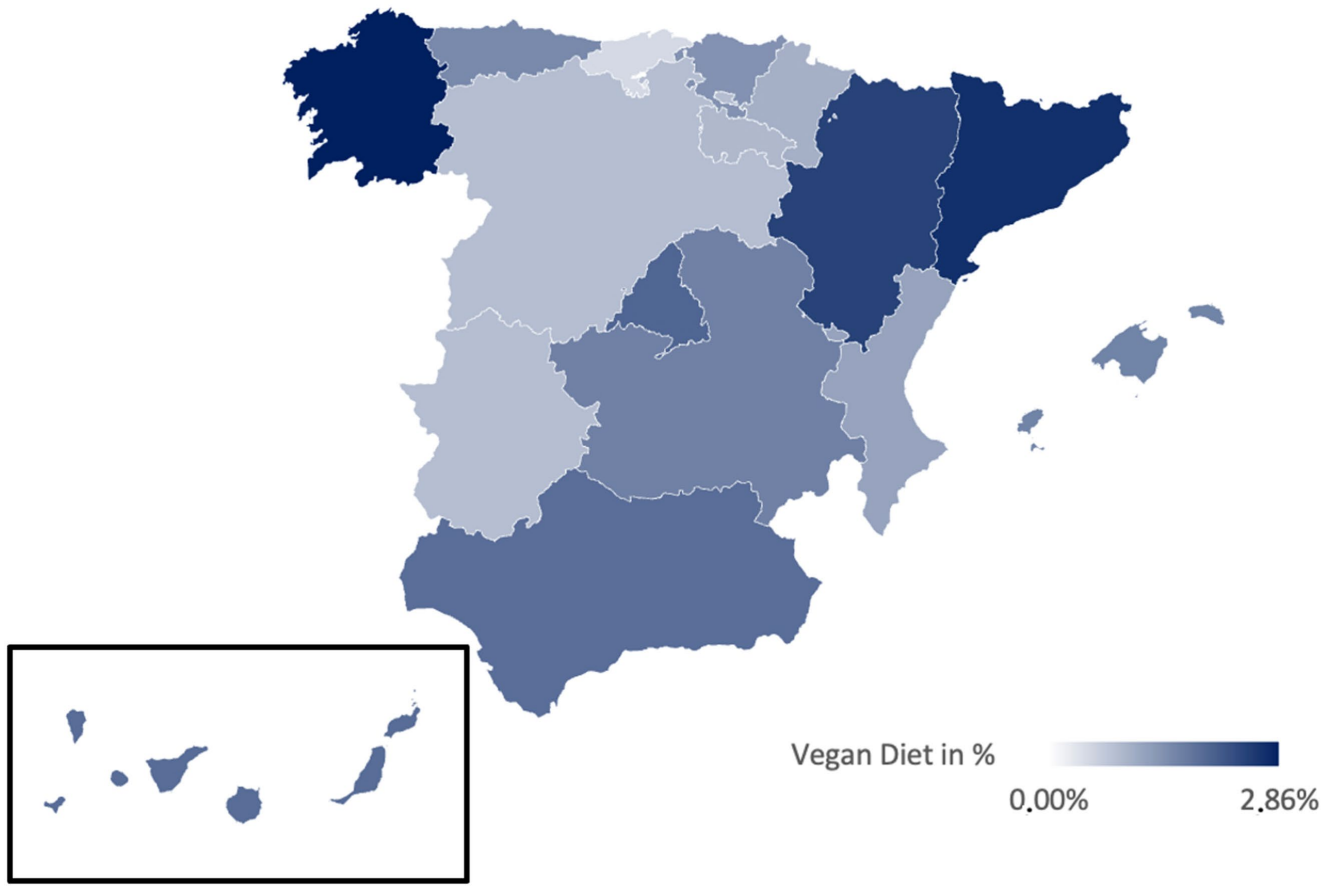
Prevalence of the Vegan diet in the different regions of Spain.

**Figure 5:**
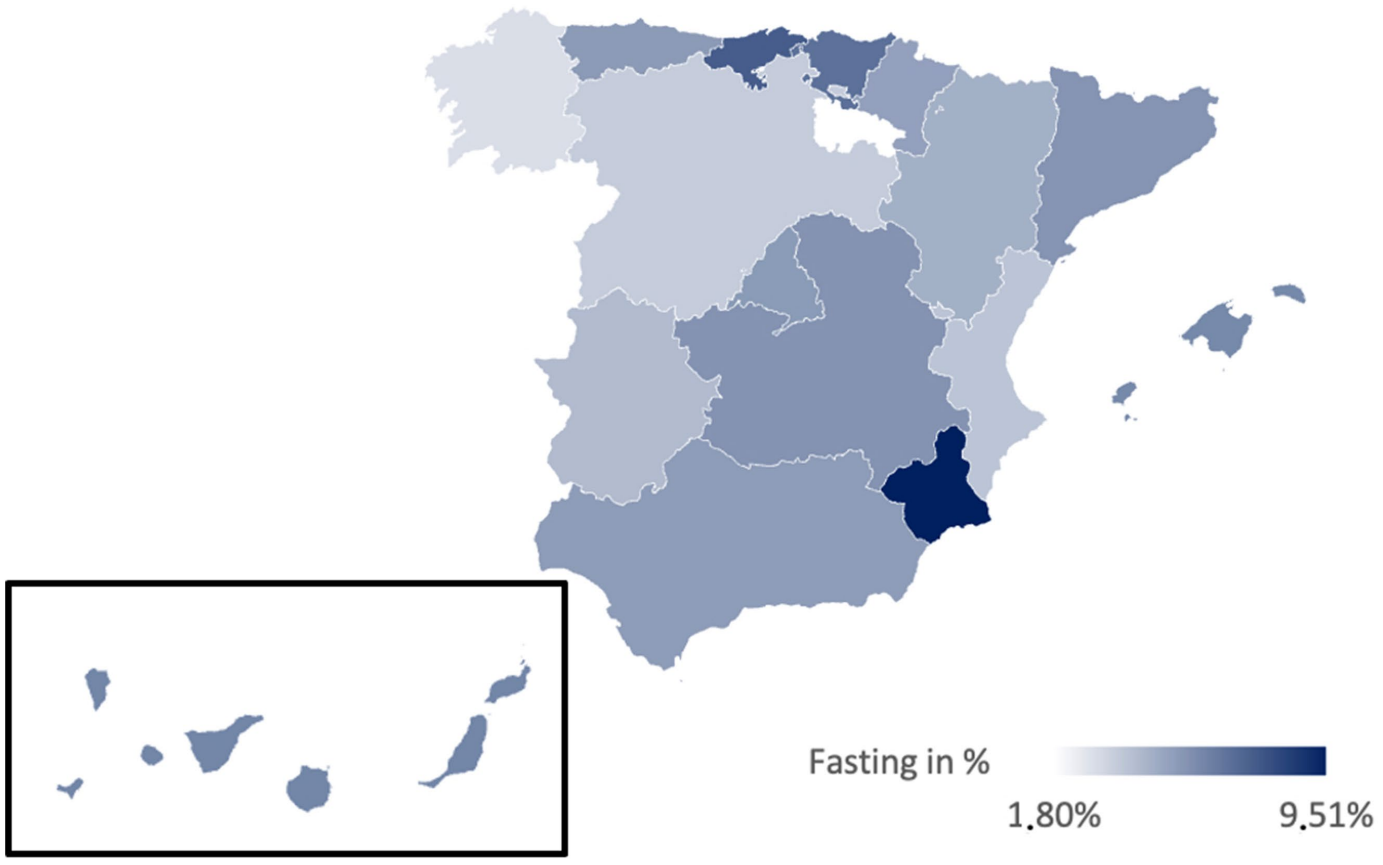
Prevalence of the intermittent fasting in the different regions of Spain.

**Figure 6:**
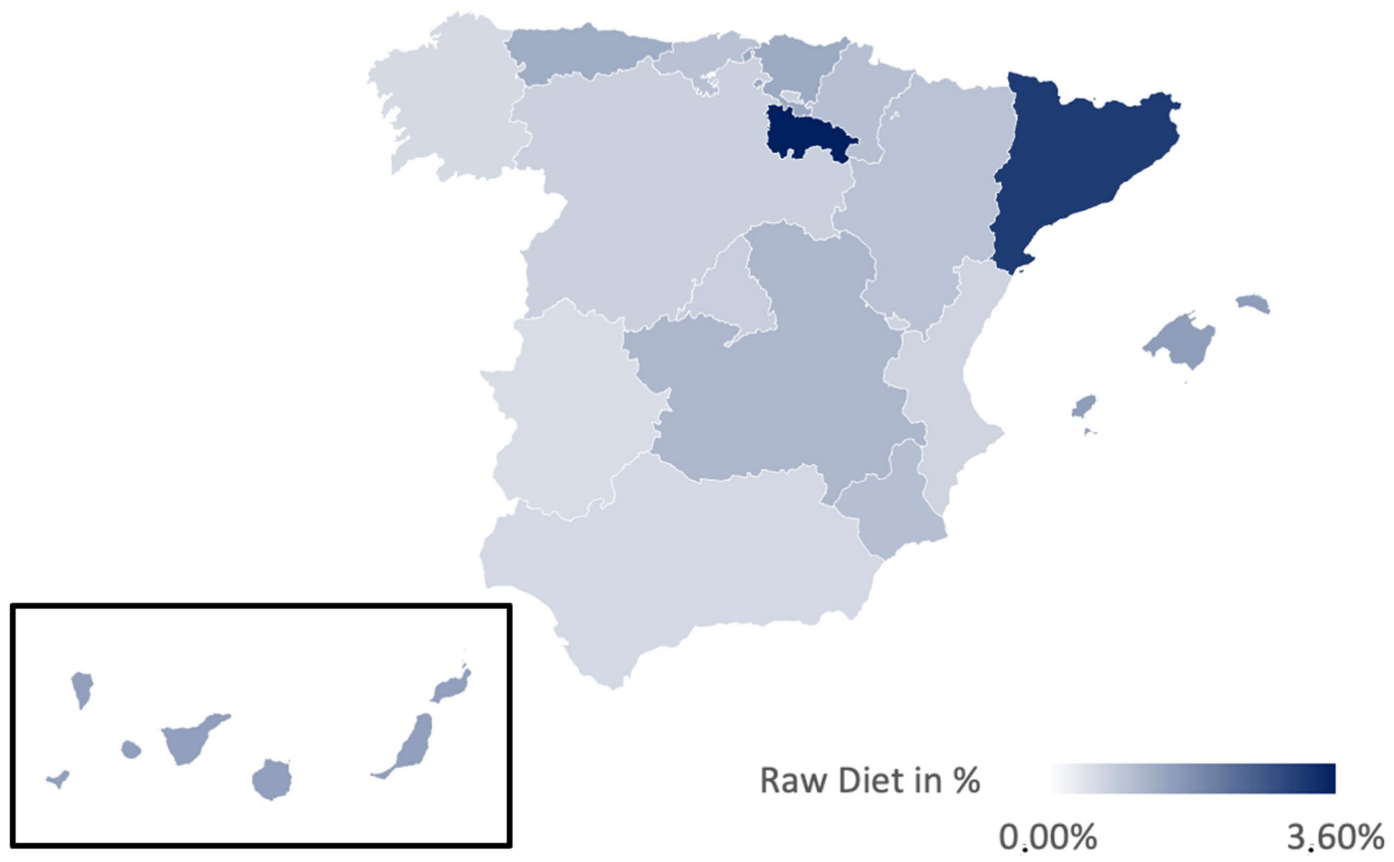
Prevalence of the raw diet in the different regions of Spain.

Table 3 presents the prevalences of various diets in Spain, illustrated in Figures 2–6. While no significant regional differences were found, trends indicate that Catalonia has, in comparison, the highest rate of vegetarians, while Extremadura presented the lowest, and in Murcia intermittent fasting was more often found than in any other region.

**Table 3:** Prevalence according to different regions of Spain.

Regions of Spain *N* (%)							
	Total (*N*)	Mediterranean diet	Vegetarian	Vegan	Fasting	Raw	*p*-value*
Mean of percentages (SD)		86.20% (4.20)	6.10% (2.51)	1.38% (0.82)	5.11% (1.74)	1.21% (0.88)	
Andalusia	2,427	2,108 (86.86%)	132 (5.44%)**	45 (1.85%)	127 (5.23%)	15 (0.62%)	*p* = 0.041***
Aragon	621	531 (85.51%)	41 (6.60%)	15 (2.42%)	28 (4.51%)	6 (0.97%)**	*p* = 0.430
Asturias	605	521 (86.12%)	35 (5.29%)	9 (1.49%)	32 (5.29%)	8 (1.32%)**	*p* = 0.186
Cantabria	203	166 (81.77%)	19 (9.36%)**	1 (0.49%)	15 (7.39%)	2 (0.99%)	*p* = 0.133
Castilla-La Mancha	689	592 (85.92%)	40 (5.81%)	11 (1.60%)	38 (5.52%)	8 (1.16%)**	*p* = 0.318
Castile and León	1,205	1,080 (89.63%)	63 (5.23%)	10 (0.83%)	43 (3.57%)**	9 (0.75%)	*p* = 0.006***
Cataluña	3,238	2,542 (78.51%)	330 (10.19%)**	86 (2.66%)	178 (5.50%)	102 (3.15%)	*p* < 0.001***
Ceuta and Melilla	26	25 (96.15%)	0 (0.00%)**	0 (0.00%)	1 (3.85%)	0 (0.00%)	*p* = 0.161
Community of Madrid	3,625	3,091 (85.27%)	243 (6.70%)	71 (1.96%)	192 (5.30%)	28 (0.77%)	—
Valencian community	3,614	3,204 (88.66%)	206 (5.70%)	42 (1.16%)	137 (3.79%)**	25 (0.69%)	*p* = 0.001***
Extremadura	361	327 (90.58%)	14 (3.88%)**	3 (0.83%)	15 (4.16%)	2 (0.55%)	*p* = 0.028***
Galicia	1,329	1,133 (85.25%)	111 (8.35%)	38 (2.86%)	39 (2.93%)**	8 (0.60%)	*p* < 0.001***
Balearic islands	443	369 (83.30%)	34 (7.67%)	7 (1.58%)	26 (5.87%)	7 (1.58%)	*p* = 0.076
Canary islands	587	485 (82.62%)	47 (8.01%)	11 (1.87%)	35 (5.96%)	9 (1.53%)**	*p* = 0.058
La Rioja	111	102 (91.89%)	2 (1.80%)	1 (0.90%)	2 (1.80%)	4 (3.60%)**	*p* = 0.003***
Navarre	297	262 (88.22%)	14 (4.71%)**	3 (1.01%)	15 (5.05%)	3 (1.01%)	*p* = 0.169
Basque country	863	713 (82.62%)	68 (7.88%)	12 (1.39%)	58 (6.72%)	12 (1.39%)**	*p* = 0.070
Region of Murcia	389	322 (82.78%)	26 (6.68%)	0 (0.00%)	37 (9.51%)**	4 (1.03%)	*p* < 0.001***

*Chi-Square test, calculated using the Community of Madrid as a fix value and the most protruding percentage in each region for the comparison, used value is marked with**. ***Tendency, but not significant, indicated by Cramer’s V. ****Significant differences, indicated by Cramer’s V.

Table 4 examines dietary prevalence between northern and southern Spain, considering climatic influences. Statistical differences were found in the appearing of vegetarianism (*p* < 0.001, Cramer’s V = 0.043), veganism (*p* = 0.003, Cramer’s V = 0.022), and raw food diets (*p* < 0.001, Cramer’s V = 0.052), with a slight inclination toward the north. However, as indicated by the minor effect sizes, these differences are trends rather than significant disparities.

**Table 4:** Prevalence in the North vs. South Spain.

	Total	Mediterranean diet	Vegetarian	Vegan	Fasting	Raw
*N* (%)						
North	9,602 (43.29%)	7,419 (42.22%)	717 (50.32%)	182 (49.86%)	436 (42.83%)	161 (63.89%)
South	12,579 (56.71%)	10,154 (57.78%)	708 (49.68%)	183 (50.14%)	582 (57.17%)	91 (36.11%)
Comparison with Mediterranean diet						
			*p* < 0.001***,	*p* = 0.003***,	*p* = 0.701,	*p* < 0.001***,
			Cramer’s V = 0.043	Cramer’s V = 0.022	Cramer’s V = 0.003	Cramer’s V = 0.052

North (Aragon, Cantabria, Castile and León, Catalonia, Chartered Community of Navarre, Galicia, Balearic Islands, La Rioja, Basque Country, Principality of Asturias) and South (Andalusia, Castilla-La Mancha, Ceuta and Melilla, Community of Madrid, Valencian Community, Extremadura, Canary Islands, Region of Murcia). Chi-Square test. ^***^Tendency, but not significant, indicated by Cramer’s V. ^****^Significant differences, indicated by Cramer’s V.

### 3.3 Relationships between socio-demographic variables and the type of diet

Table 5 illustrates the relationships between socio-demographic variables and dietary choices. It was found that women are more likely to adopt PBDs such as the vegetarian diet (*p* < 0.001, Cramer’s V = 0.0484), the vegan diet (*p* = 0.004, Cramer’s V = 0.0214), and the raw food diets (*p* = 0.008, Cramer’s V = 0.0197), while men tend to practice intermittent fasting (IF) more than women (*p* = 0.009, Cramer’s V = 0.0191).

**Table 5:** Relationships between the type of diet followed, excluding the group “Others,” and socio-demographic variables (*N* = 20,633).

Total	Mediterranean Diet	Vegetarian	Vegan	Fasting	Raw
Sex					
Male [3,963 (19.20%)]	3,467 (19.73%)	178 (12.49%)	50 (13.70%)	235 (23.08%)	33 (13.10%)
Female [16,670 (80.80%)]	14,106 (80.27%)	1,247 (87.51%)	315 (86.30%)	783 (76.92%)	219 (86.90%)
Vegetarian	***p < 0.001, Cramer’s V = 0.0484				
Vegan	***p = 0.004, Cramer’s V = 0.0214	*p* = 0.537, Cramer’s V = 0.0146			
Fasting	***p = 0.009, Cramer’s V = 0.0191	***p < 0.001, Cramer’s V = 0.0139	***p < 0.001, Cramer’s V = 0.102		
Raw	***p = 0.008, Cramer’s V = 0.0197	*p* = 0.790. Cramer’s V = 0.00651	*p* = 0.829,	***p < 0.001, Cramer’s V = 0.0976	
			Cramer’s V = 0.00869		
Age					
18–25 years [5,697 (27.64%)]	4,829 (27.48%)	553 (38.81%)	134 (36.71%)	179 (17.58%)	20 (7.94%)
26–40 years [8,923 (43.28%)]	7,479 (42.56%)	647 (45.40%)	168 (46.03%)	483 (47.45%)	146 (5794%)
41–65 years [5,819 (28.23%)]	5,096 (29.00%)	222 (15.57%)	63 (17.26%)	352 (34.58%)	86 (34.13%)
>65 years [176 (0.85%)]	169 (0.96%)	3 (0.21%)	0 (0.00%)	4 (0.39%)	0 (0.00%)
Vegetarian	***p < 0.001, Cramer’s V = 0.0909				
Vegan	***p < 0.001, Cramer’s V = 0.0426	*p* = 0.650. Cramer’s V = 0.0303			
Fasting	***p < 0.001, Cramer’s V = 0.0539	***p < 0.001, Cramer’s V = 0.273	***p < 0.001, Cramer’s V = 0.288		
Raw	***p < 0.008, Cramer’s V = 0.0524	***p < 0.001, Cramer’s V = 0.253	****p < 0.001, Cramer’s V = 0.340	***p < 0.001, Cramer’s V = 0.117	

Education level					
Low/Medium [6,461 (31.31%)]	5,528 (31.46%)	443 (31.09%)	127 (34.79%)	294 (28.88%)	69 (27.38%)
High [14,172 (68.69%)]	12,045 (68.54%)	982 (68.91%)	238 (65.21%)	724 (71.12%)	183 (72.62%)
Vegetarian	*p* = 0.773,				
	Cramer’s V = 0.00210				
Vegan	*p* = 0.174,	*p* = 0.175, Cramer’s V = 0.0321			
	Cramer’s V = 0.0101				
Fasting	*p* = 0.085,	*p* = 0.241, Cramer’s V = 0.0237	***p = 0.035,		
	Cramer’s V = 0.0126		Cramer’s V = 0.0566		
Raw	*p* = 0.166,	*p* = 0.239, Cramer’s V = 0.0288	*p* = 0.052,	*p* = 0.637 Cramer’s V = 0.0132	
	Cramer’s V = 0.0104		Cramer’s V = 0.0783		
Income level					
Low/ Medium [9,009 (47.61%)]	7,549 (46.89%)	703 (54.37%)	204 (60.71%)	446 (46.65%)	107 (44.40%)
High [9,915 (52.39%)]	8,549 (53.11%)	590 (45.63%)	132 (39.29%)	510 (53.35%)	134 (55.60%)
Vegetarian	***p < 0.001, Cramer’s V = 0.0383				
Vegan	***p < 0.001, Cramer’s V = 0.0377	*p* = 0.082, Cramer’s V = 0.0529			
Fasting	***p = 0.034, Cramer’s V = 0.0191	***p < 0.001, Cramer’s V = 0.0935	***p < 0.001,		
			Cramer’s V = 0.123		
Raw	*p* = 0.052,	***p < 0.001, Cramer’s V = 0.0945	***p < 0.001,	*p* = 0.471 Cramer’s V = 0.0345	
	Cramer’s V = 0.0182		Cramer’s V = 0.172		
Municipality					
<2000 inhabitants [938 (4.55%)]	796 (4.53%)	60 (4.21%)	20 (5.48%)	41 (4.03%)	21 (8.33%)
2000–10,000 inhabitants [3,305 (16.02%)]	2,800 (15.93%)	229 (16.07%)	56 (15.34%)	169 (16.60%)	51 (20.24%)
>10,000 inhabitants [16,390 (79.44%)]	13,977 (79.54%)	1,136 (79.72%)	289 (79.18%)	808 (7.37%)	180 (71.43%)
Vegetarian	*p* = 0.853,				
	Cramer’s V = 0.00410				
Vegan	*p* = 0.673,	*p* = 0.562, Cramer’s V = 0.0254			
	Cramer’s V = 0.00665				
Fasting	*p* = 0.667,	*p* = 0.923, Cramer’s V = 0.00809	*p* = 0.461,		
	Cramer’s V = 0.00660		Cramer’s V = 0.0334		
Raw	***p = 0.002, Cramer’s V = 0.0268	***p = 0.003, Cramer’s V = 0.0833	*p* = 0.079,	***p = 0.004 Cramer’s V = 0.0924	
			Cramer’s V = 0.0906		

Chi Square test. ^***^Tendency, but not significant as indicated by Cramer’s V. ****Significant differences, indicated by Cramer’s V.

Younger individuals (18–25 years) are more likely to follow vegetarian (9.2 vs. 6.4% overall) and vegan diets (2.2 vs. 1.6% overall), but less likely to engage in IF (3.0 vs. 4.6% overall) or raw food diets (0.34 vs. 1.1% overall). In contrast, older adults (41–65 years) show higher adherence to IF (5.51%) and raw food diets (1.35%). Comparisons between the dietary groups and the MD group regarding the age reveal tendencies, but no statistically significant differences, nonetheless comparing the diets with each other showed that raw foodists are significantly older than vegans (*p* < 0.001, Cramer’s V = 0.340) and that IF practitioners are generally older than those following other diets (*p* < 0.001).

The education level didn’t seem the influence the dietary choice at all. However, individuals with lower income levels are more likely to adopt the vegetarian (*p* < 0.001, Cramer’s V = 0.0383) and the vegan diet (*p* < 0.001, Cramer’s V = 0.0377). Additionally, people living in villages show a higher tendency to practice raw food diets (*p* = 0.002, Cramer’s V = 0.0268).

### 3.4 Relationships between habits variables and health variables and the type of diet

Table 6 presents the relationships between the diet types and various nutrition and lifestyle habits, and the health variables, including the BMI and the self-perceived health. No significant differences were found regarding coffee consumption (*p* = 0.531), self-perceived health (*p* = 0.865), sleep quality (*p* = 0.291), or the frequency of getting drunk (*p* = 0.094). However, trends emerged, albeit weakly, for variables like BMI, sedentary lifestyle, and hours of sleep. Significant differences were noted in the IASE scores for vegetarians (*p* < 0.001, *r* = 0.323), vegans (*p* < 0.001, *r* = 0.752), and raw foodists (*p* < 0.001, *r* = 0.752) compared to the MD practitioners (Table 7).

**Table 6:** Relationship between habits variables in five different groups of population (vegetarian, vegan, Mediterranean, those eating an unprocessed diet, and those practicing intermittent fasting).

	Mediterranean diet	Vegetarian	Vegan	Fasting	Raw	*p-*value*
Health values Mean (SD)						
BMI	24.00 (4.31)	22.50 (3.65)	21.90 (3.60)	23.20 (4.15)	22.50 (3.60)	*p* < 0.001^†^
IASE	56.30 (9.49)	53.50 (7.36)	42.50 (5.85)	53.80 (10.10)	43.80 (9.76)	*p* < 0.001^††^
Fried food consumption	2.33 (0.81)	2.00 (0.75)	1.96 (0.75)	1.96 (0.79)	1.69 (0.72)	*p* < 0.001^†^
Fast food consumption	2.44 (0.75)	2.25 (0.77)	2.28 (0.76)	2.16 (0.74)	1.66 (0.72)	*p* < 0.001^†^
Ultra-processed food consumption	2.39 (0.95)	2.18 (0.90)	2.13 (0.82)	2.06 (0.88)	1.54 (0.69)	*p* < 0.001^†^
Water consumption	3.38 (0.64)	3.45 (0.62)	3.43 (0.60)	3.52 (0.60)	3.49 (0.51)	*p* < 0.001^†^
Soft drink consumption	1.43 (0.69)	1.21 (0.51)	1.20 (0.48)	1.28 (0.58)	1.08 (0.27)	*p* < 0.001^†††^
Juice consumption	1.26 (0.55)	1.19 (0.47)	1.22 (0.52)	1.16 (0.47)	1.10 (0.39)	*p* < 0.001^†^
Coffee consumption	1.72 (0.71)	1.66 (0.66)	1.56 (0.68)	1.75 (0.71)	1.65 (0.75)	*p* = 0.531
Self-perceived health	3.82 (0.82)	3.88 (0.81)	3.91 (0.92)	3.87 (0.83)	3,66 (0.94)	*p* = 0.865
Sedentary lifestyle	1.58 (0.84)	1.62 (0.85)	1.61 (0.87)	1.51 (0.77)	1,42 (0.75)	*p* < 0.001^†^
Exercise (min/week)	145 (171.00)	190 (180.00)	214 (213)	232 (190)	194 (155)	*p* < 0.001^†††^
Sleeping hours	2.51 (0.73)	2.60 (0.72)	2.55 (0.74)	2.55 (0.74)	2.80 (0.65)	*p* < 0.001^†^
Getting up rested	2.54 (0.58)	2.54 (0.56)	2.53 (0.63)	2.60 (0.59)	2.56 (0.59)	*p* = 0.500
Sleeping quality	3.39 (1.02)	3.40 (1.02)	3.38 (1.11)	3.44 (0.96)	3.48 (1.00)	*p* = 0.291
Smoking	1.25 (0.66)	1.13 (0.46)	1.11 (0.39)	1.17 (0.55)	1.12 (0.40)	*p* < 0.001^†^
Alcohol consumption	1.80 (0.89)	1.58 (0.76)	1.50 (0.75)	1.70 (0.83)	1.44 (0.69)	*p* < 0.001^†††^
Getting drunk	1.06 (0.30)	1.06 (0.26)	1.02 (0.26)	1.06 (0.26)	1.03 (0.22)	*p* = 0.094
Nightlife	1.20 (0.44)	1.18 (0.41)	1.10 (0.32)	1.15 (0.39)	1.11 (0.32)	*p* < 0.001^†^
Obesophobia	3.42 (1.43)	3.48 (1.41)	3.40 (1.42)	3.68 (1.42)	2.91 (1.40)	*p* < 0.001^†^
No control	2.75 (2.84)	2.84 (1.25)	2.67 (1.34)	2.93 (1.26)	2.53 (1.18)	*p* < 0.001^†^
Body image	3.57 (1.28)	3.69 (1.28)	3.69 (1.31)	3.82 (1.32)	3.22 (1.15)	*p* < 0.001^†^

Mann–Whitney *U* test. Comparison between the Mediterranean Diet group and the special diets groups. ^†^Tendencies noticeable, but not significant as shown by effect size *r* < 0.3. ^††^Significant differences indicated by effect size *r* > 0.3. ^†††^Tendencies not significant, but big differences between groups and for that interesting for deeper analysis.

**Table 7:** Analysis of the relationship between the chosen habit variables and the different types of diets that show significant differences indicated by the effect size or big differences between groups.

	Mediterranean diet	Vegetarian	Vegan	Fasting	Raw
IASE					
Vegetarian	*p* < 0.001^††^, *r* = 0.323				
Vegan	*p* < 0.001^††^, *r* = 0.752	*p* < 0.001^††^, *r* = 0.684			
Fasting	*p* < 0.001^†^, *r* = 0.152	*p* < 0.001^†^, *r* = 0.142	*p* < 0.001^††^, *r* = 0.629		
Raw	*p* < 0.001^††^, *r* = 0.752	*p* < 0.001^††^, *r* = 0.451	*p* < 0.001^†^, *r* = 0.093	*p* < 0.001^††^, *r* = 0.464	
Soft drink consumption					
Vegetarian	*p* < 0.001^†^, *r* = 0.159				
Vegan	*p* < 0.001^†^, *r* = 0.165	*p* = 0.831, *r* = 0.005			
Fasting	*p* < 0.001^†^, *r* = 0.111	*p* = 0.003^†^, r < 0.001	*p* = 0.032^†^, *r* = 0.054		
Raw	*p* < 0.001^†^, *r* = 0.263	*p* < 0.001^†^, *r* = 0.104	*p* < 0.001^†^, *r* = 0.099	*p* < 0.001^†^, *r* = 0.152	
Alcohol consumption					
Vegetarian	*p* < 0.001^†^, *r* = 0.126				
Vegan	*p* < 0.001^†^, *r* = 0.193	*p* = 0.016^†^, *r* = 0.072			
Fasting	*p* = 0.001^†^, *r* = 0.056	*p* < 0.001^†^, *r* = 0.070	*p* < 0.001^†^, *r* = 0.139		
Raw	*p* < 0.001^†^, *r* = 0.225	*p* = 0.003^†^ *r* = 0.104	*p* = 0.427, *r* = 0.032	*p* < 0.001^†^, *r* = 0.172	
Exercise (h/week)					
Vegetarian	*p* < 0.001^†^, *r* = 0.171				
Vegan	*p* < 0.001^†^, *r* = 0.189	*p* = 0.324 *r* = 0.033			
Fasting	*p* < 0.001^††^, *r* = 0.301	*p* < 0.001^†^, *r* = 0.137	*p* = 0.008^†^, *r* = 0.092		
Raw	*p* < 0.001^†^, *r* = 0.234	*p* = 0.301 *r* = 0.040	*p* = 0.917, *r* = 0.005	*p* = 0.005^†^, *r* = 0.114	

Mann–Whitney *U* test. ^†^Tendencies noticeable, but not significant as shown by effect size *r* < 0.3. ^††^Significant differences indicated by effect size *r* > 0.3.

The analysis showed that plant-based dieters, raw foodists and those practicing IF consume sugary drinks and alcohol less frequently compared to MD followers. Notably, vegetarians and vegans showed a lower frequency of sugary drink consumption (*p* < 0.001, *r* = 0.159; *p* < 0.001, *r* = 0.165, respectively) and alcohol (*p* < 0.001, *r* = 0.126; *p* < 0.001, *r* = 0.193). In terms of physical activity, plant-based dieters, IF followers and raw foodists tended to engage in more exercise per week (Vegetarian: 190 min; Vegan: 214 min; IF: 232 min; Raw: 194 min) compared to the MD followers (145 min).

Table 8 discusses the prevalence of eating disorders in relation to the diet type, indicating a slight tendency for individuals following non-MD diets to experience these disorders (Vegetarian *p* < 0.001, Cramer’s V = 0.061; Vegan *p* < 0.001, Cramer’s V = 0.029; IF *p* < 0.001, Cramer’s V = 0.045; Raw *p* < 0.001, Cramer’s V = 0.027), though these trends are weak and not statistically significant.

**Table 8:** Relationship between having a diagnosed eating disorder in five different groups of population (vegetarian, vegan, Mediterranean, those eating an unprocessed diet, and those practicing intermittent fasting).

*N* (%)						
	Total	Mediterranean Diet	Vegetarian	Vegan	Fasting	Raw
Diagnosed ED						
Diagnosed ED	780 (3.52%)	471 (2.68%)	91 (6.40%)	29 (7.95%)	49 (4.81%)	16 (6.35%)
No diagnosed ED	21,401 (96.48%)	17,102 (97.32%)	1,331 (90.60%)	336 (92.05%)	969 (95.19%)	236 (93.65%)
Comparison with Mediterranean diet						
			*p* < 0.001***, Cramer’s V = 0.061	*p* < 0.001***, Cramer’s V = 0.029	*p* < 0.001***, Cramer’s V = 0.045	*p* < 0.001***, Cramer’s V = 0.027

Chi Square test. ^***^Tendency, but not significant as indicated by Cramer’s V.

## 4 Discussion

The study reveals that in Spain still 79.2% of the population adheres to a Mediterranean diet, historically the dominant dietary style (43). However, there is a noticeable shift toward alternative diets, with 6.4% following a vegetarian diet and 1.6% a vegan diet, totaling 8% adopting some form of plant-based diet (PBD) (7, 8). Intermittent fasting (IF) is practiced by 4.6% of respondents, a choice that has gained popularity due to its perceived health benefits, especially for weight loss (44–47). A small minority, 1.1%, follows a raw food diet, while 7% adhere to other dietary patterns, indicating a diversification of choices in the Spanish populations (48, 49).

Regionally, the Mediterranean diet is most prevalent in La Rioja (91.89%) and least in Catalonia (78.51%) (Figure 2). The vegetarian diet is more prominent in Galicia, Cantabria, and Catalonia, while La Rioja and Extremadura presented lower percentages (Figure 3). The vegan diet is most common in Galicia, Catalonia, and Aragon, with Cantabria having the lowest prevalence (Figure 4). For intermittent fasting, the Region of Murcia leads at 9.51%, while La Rioja and Galicia exhibit very low rates (Figure 5). Lastly, the raw food diet has the highest prevalence in La Rioja and Catalonia, with other regions showing generally low rates (Figure 6).

The prevalence of dietary choices in Spain varies due to regional, cultural, economic, and environmental factors that influences the food preferences (50). Differences in the plant-based diet prevalence between regions like Catalonia and Galicia versus La Rioja and Extremadura may stem from the regional gastronomic culture, as both Catalonia and Galicia have culinary traditions featuring diverse plant-based dishes. For instance, Catalan cuisine includes dishes like “escalivada” and “pa amb tomàquet” (51), while Galicia focuses on fresh seafood and seasonal vegetables (52).

Moreover, environmental and ethical awareness may also drive PBD adoption, with certain regions exhibiting greater concern for sustainability and animal welfare (53, 54). Geographic factors, such as Galicia’s coastal proximity and Catalonia’s diversity, facilitate access to fresh products, making vegetarian and vegan diets more attainable (51, 52). In contrast, La Rioja’s and Extremadura’s strong meat and dairy traditions may hinder the acceptance of PBDs (55, 56).

Regarding the higher prevalence of IF in Murcia, this trend lacks a definitive explanation. The region’s rich culinary heritage may promote awareness of balanced diets, making individuals more receptive to IF as a healthy practice (57). Additionally, sampling bias could exist if influencers promoting intermittent fasting were from Murcia, potentially skewing survey results toward higher adherence in that region.

The study shows that women tend to adopt more often than men the vegetarian and the vegan diet, and raw food diets. This aligns with previous studies, including one in Switzerland, which indicated that pescatarians and flexitarians are more likely to be female, and another study in France found that vegetarians are often female, younger, and self-employed (58, 59). Several factors may explain this trend. Women are generally more health-conscious and proactive in adopting health-promoting behaviors (60, 61). PBDs are perceived as healthier due to their emphasis on nutrient-rich foods (62). Additionally, social media exposure may influence dietary choices, as women might encounter more pro-plant-based messaging online (63). Ethical and environmental considerations also play a role, with women more likely to adopt diets perceived as sustainable and ethical (64, 65).

Conversely, men are more inclined to practice IF than women. Literature on IF prevalence among genders is mixed; while some studies show a male prevalence, others indicate that women may engage in it for a longer period of time (66, 67). Men’s inclination toward IF can be linked to interests in fitness, bodybuilding, and weight loss. It is popular among men seeking to lose body fat while maintaining muscle mass (68, 69). Moreover, men may have a greater focus on physical appearance, making IF appealing as a method for weight control and muscle definition (70, 71).

The data indicates that younger individuals (ages 18–25) tend to prefer vegetarian (9.29%) and vegan (2.25%) diets over intermittent fasting (3.0%) and raw food diets (0.34%), which are more common among older adults. This aligns with previous research highlighting a higher prevalence PBDs among the youth (72, 73). Several hypotheses explain this trend: younger people may be more concerned about environmental issues and animal welfare (64, 74), have greater access to information about vegetarian and vegan diets via the internet and social media (75, 76), and are generally more open to experimenting with different lifestyles (77). On the other hand, IF (5.51%) and raw food diets (1.35%) are more frequently practiced by older adults (ages 41–65), although differences compared to the Mediterranean diet are not significant. Middle-aged individuals are often more focused on health and wellbeing and seek dietary strategies for improvement, especially as aging can lead to metabolic changes and weight gain (78). IF is linked to various health benefits (79), which may motivate adherence among this group. Additionally, some middle-aged individuals might find IF easier to manage due to decreased appetite or energy mobilization (80), along with a more stable lifestyle that allows for better scheduling of fasting practices.

The analysis of education level revealed no differences in dietary choices among the explored diets, which contradicts existing literature that indicates higher education positively influences dietary choices and habits (81–83). Many studies have linked higher educational attainment with greater adoption of PBDs (58, 59). The lack of differences may be due to the sample characteristics, as a majority (68.3%) possess higher education and are predominantly young (ages 18–25: 26.8%; 25–40: 43.5%). Future research could focus on older, less educated populations to better understand this relationship.

Regarding income level, individuals with lower income levels showed a greater tendency to follow vegetarian and vegan diets. Previous literature supports the link between income and diet, noting that lower income often correlates with less healthy diets (84–86). However, some studies suggest that PBDs are associated with lower incomes, as demonstrated by Wozniak et al. (58), which found that vegetarians often had lower incomes than omnivores. Several factors may explain this relationship: plant-based foods can be more affordable than animal products (87), health concerns may drive lower-income individuals to adopt vegetarian or vegan diets to mitigate chronic disease risks (88, 89), and ethical considerations might motivate some to choose these diets as a form of solidarity with less privileged communities.

An interesting tendency to adopt a raw food diet was observed among individuals living in villages compared to city dwellers. This may be attributed to the closer connection rural residents have to their food sources, often engaging in food production or gardening, which can inspire adherence to diets focused on fresh, minimally processed ingredients like raw vegan diets. Additionally, those in rural areas may have better access to fresh fruits, vegetables, and raw ingredients through local farmers’ markets or community gardens, making it easier to follow a raw food diet.

Examining the IASE score reveals that plant-based dieters have lower scores compared to the MD followwers, with vegan diets scoring particularly low (14 points lower) and raw food diets even lower (23 points lower). This disparity may arise from the IASE index being based on a varied diet that includes animal proteins such as meat and dairy (39). Since vegan and raw vegan diets exclude these foods, their IASE scores are significantly lower, limiting the index’s reliability in reflecting the health benefits of these diets. Notably, across all groups, including the Mediterranean diet, the nutritional index was found within the “Needs changes” range (36.5 ≤ IASE ≤ 58.4), indicating a general deficiency in the nutrition of the Spanish population, as highlighted in several recent studies (90–92).

The analysis reveals that individuals consuming plant-based diets, including raw food and vegan diets, exhibit lower consumption of sugary drinks and alcohol compared to those following the MD. This is particularly evident in alcohol consumption, which aligns with the for the MD typical inclusion of wine (93), while reduced intake of sugar-sweetened beverages may result from calorie-restriction tendencies among people practicing any other form of diet (94).

Furthermore, those practicing IF engage in significantly more weekly physical activity compared to other diet groups, especially the Mediterranean diet. This aligns with the motivation of intermittent fasters to manage weight, as exercise is a critical component of weight control (12). Individuals adhering to non-Mediterranean diets also show a greater commitment to health and fitness, reflected in their weekly exercise durations, which exceed the World Health Organization’s recommendation of 150 min for maintaining a healthy lifestyle (95).

Lastly, the presence of diagnosed eating disorders (ED) shows a slight correlation with following diets outside the standard Mediterranean approach. This trend supports the notion that dieting can be a triggering factor for the development of eating disorders (96).

### 4.1 Strengths and limitations

This study possesses notable strengths, with the foremost being its substantial sample size (*N* = 22,181), offering a comprehensive portrayal of the health behaviors among the Spanish population. Additionally, the study benefits from the geographical inclusivity of its sample, encompassing data from all regions of Spain, including both urban and rural areas, as well as the canary and balneary islands. Another strength lies in the extensive range of variables investigated, enabling an examination of the interplay between various nutritional, social and lifestyle factors as well as physical activity with socio-demographic determinants, thereby providing insights into the health status of the Spanish people.

However, the study does have its limitations. Data collection through an online survey method, while facilitating access to the target population, may introduce response bias. Efforts have been made to mitigate the possibility of erroneous or misleading responses by using a closed and anonymous questionnaire format. In addition, a significant effort has been made to disseminate the questionnaire also through physical means that do not involve dissemination through social networks. Self-report bias is a significant concern in cohort studies using nutritional surveys. This type of bias can arise when participants provide inaccurate or biased information about their food consumption and dietary habits. Although this limitation is intrinsic and difficult to overcome, it is encouraging to note that the results of this study are largely in line with findings highlighted in previous research. This concordance provides confidence in the conclusions reached through this study. A gender bias was also observed, with 80.8% of survey respondents being female. This finding is consistent with existing research indicating that males are less inclined to participate in surveys (97, 98). To mitigate this gender bias as much as possible, concerted efforts were made to recruit male participants for the study. Despite challenges in achieving parity with female participation, a substantial sample of 4,251 males was successfully obtained. A final limitation of the study is that it investigated the people who practice Intemittent Fasting and the relationship with lifestyle and health habits, but it did not study the type of IF or the duration and frequency of IF. On the other hand, it should also be considered that IF was considered as a type of diet and this is a limitation, because some people might practice both IF and the other specific diets investigated.

### 4.2 Areas for further research

We believe that this study represents the starting point for future research in this field and that it could be interesting to investigate the relationship between the type of diet adopted and habits in children and adolescents, and not only in adults, as it has been done so far. It may also provide helpful information for disseminating the questionnaire, specifically focusing on a male sample.

Another possible future development could be to extend this study to other countries, analyzing whether the conclusions obtained for the Spanish population can be extrapolated to other regions or if significant differences can be observed. In this sense, the authors are in the process of translating and culturally adapting the instrument for the Italian and Chilean population and will soon be able to collect data in those countries as well.

Further investigation, which could undoubtedly be of interest, is to analyze the culinary habits of the population in relation to the type of diet adopted. The aim would be to see if there are significant differences between people who spend time preparing and eating a home-cooked meal and those who, due to lack of time, motivation or knowledge, do not cook and frequently resort to fourth and fifth range products or pre-cooked dishes. It would also be interesting to know where people look for and find recipes and advice on how to prepare dishes.

## 5 Conclusion

Among the findings of this study, younger people show a higher propensity to adopt a PBD compared to older age groups, while women tend to follow this type of diet more often than men. On the other hand, men seem to practice intermittent fasting more than women. In addition, a greater tendency to follow a raw food diet is identified among residents of rural areas compared to urban dwellers. Regional variations are also observed in Spain, with a higher adoption of PBDs in Catalonia, while intermittent fasting is more common in the Region of Murcia. Numerous studies have demonstrated the healthfulness of the Mediterranean dietary pattern, and its importance is undeniable, however, our results show that people who engage in other dietary patterns than Mediterranean diet, are more health-conscious, which translates into the adoption of healthier habits, such as increased physical exercise and reduced consumption of alcohol and sugary drinks.

The results obtained in this study contribute significantly to broaden the understanding of the dietary patterns of the Spanish population and how these are influenced by sociodemographic factors. Furthermore, they provide new evidence on the impact of the adopted diet on social habits, lifestyle and health of the population.

These results are particularly relevant because they allow the comparison to the Mediterranean diet, which has historically been widely adopted by the Spanish population, with other less known and less studied dietary patterns, such as PBDs or IF, which became increasingly prevalent in the population in recent years, so gaining evidence on their impact on health is of utmost importance in the field of public health.

## Data Availability

The raw data supporting the conclusions of this article will be made available by the authors, without undue reservation.

## References

[1] Cena H Calder PC. Defining a healthy diet: evidence for the role of contemporary dietary patterns in health and disease. Nutrients. (2020) 12:334. doi: 10.3390/NU12020334, PMID: 32012681 PMC7071223

[2] Firth J Gangwisch JE Gangwisch JE Borisini A Wootton RE Wootton RE. Food and mood: how do diet and nutrition affect mental wellbeing? BMJ. (2020) 369:2382. doi: 10.1136/BMJ.M2382, PMID: 32601102 PMC7322666

[3] Bartrina JA Rodrigo CP Aresti GA Moreiras GV Serra-Majem L. Controversies about population, clinical or basic research studies related with food, nutrition, physical activity and lifestyle. Nutr Hosp. (2015) 31:15–21. doi: 10.3305/NH.2015.31.SUP3.8746 25719766

[4] Guasch-Ferré M Willett WC. The Mediterranean diet and health: a comprehensive overview. J Intern Med. (2021) 290:549–66. doi: 10.1111/JOIM.13333, PMID: 34423871

[5] Shen J Wilmot KA Ghasemzadeh N Molloy DL Burkman G Mekonnen G. Mediterranean dietary patterns and cardiovascular health. Annu Rev Nutr. (2015) 35:425–49. doi: 10.1146/ANNUREV-NUTR-011215-025104 25974696

[6] Dominguez LJ Veronese N Di Bella G Cusumano C Parisi A Tagliaferri F. Mediterranean diet in the management and prevention of obesity. Exp Gerontol. (2023) 174:112121. doi: 10.1016/J.EXGER.2023.112121 36792040

[7] Alemán JA Rentero MPZ Montoro-García S Mulero J Garrido AP Leal M. Adherence to the “Mediterranean diet” in Spain and its relationship with cardiovascular risk (DIMERICA study). Nutrients. (2016) 8:680. doi: 10.3390/NU8110680, PMID: 27801819 PMC5133068

[8] Herrera-Ramos E Tomaino L Sánchez-Villegas A Ribas-Barba L Gómez SF Wärnberg J. Trends in adherence to the Mediterranean diet in Spanish children and adolescents across two decades. Nutrients. (2023) 15:2348. doi: 10.3390/NU15102348, PMID: 37242233 PMC10223797

[9] Yokoyama Y Nishimura K Barnard ND Takegami M Watanabe M Sekikawa A. Vegetarian diets and blood pressure: a meta-analysis. JAMA Intern Med. (2014) 174:577–87. doi: 10.1001/JAMAINTERNMED.2013.14547 24566947

[10] Kahleova H Levin S Barnard ND. Vegetarian dietary patterns and cardiovascular disease. Prog Cardiovasc Dis. (2018) 61:54–61. doi: 10.1016/J.PCAD.2018.05.002 29800598

[11] Tantamango-Bartley Y Knutsen SF Knutsen R Jacobsen BK Fan J Lawrence Beeson W. Are strict vegetarians protected against prostate cancer? Am J Clin Nutr. (2016) 103:153–60. doi: 10.3945/AJCN.114.106450, PMID: 26561618 PMC4691666

[12] Welton S Minty R O’Driscoll T Willms H Poirier D Madden S. Intermittent fasting and weight loss: systematic review. Can Fam Physician. (2020) 66:117–25. PMID: 32060194 PMC7021351

[13] Yuan X Wang J Yang S Gao M Cao L Li X. Effect of intermittent fasting diet on glucose and lipid metabolism and insulin resistance in patients with impaired glucose and lipid metabolism: a systematic review and meta-analysis. Int J Endocrinol. (2022) 2022:1–9. doi: 10.1155/2022/6999907, PMID: 35371260 PMC8970877

[14] Varady KA Cienfuegos S Ezpeleta M Gabel K. Cardiometabolic benefits of intermittent fasting. Annu Rev Nutr. (2021) 41:333–61. doi: 10.1146/ANNUREV-NUTR-052020-041327 34633860

[15] Gudden J Arias Vasquez A Bloemendaal M. The effects of intermittent fasting on brain and cognitive function. Nutrients. (2021) 13:3166. doi: 10.3390/NU13093166, PMID: 34579042 PMC8470960

[16] Longo VD Di Tano M Mattson MP Guidi N. Intermittent and periodic fasting, longevity and disease. Nat Aging. (2021) 1:47–59. doi: 10.1038/S43587-020-00013-3, PMID: 35310455 PMC8932957

[17] Ivanova S Delattre C Karcheva-Bahchevanska D Benbasat N Nalbantova V Ivanov K. Plant-based diet as a strategy for weight control. Food Secur. (2021) 10:23052. doi: 10.3390/FOODS10123052, PMID: 34945602 PMC8701327

[18] Going Plant-Based: The Rise of Vegan and Vegetarian Food | Eur Secur. Available at: https://go.euromonitor.com/sb-packaged-food-210330-rise-vegan-vegetarian-food.html (Accessed March 12, 2024).

[19] Acevedo Cantero P Ortega Santos CP López-Ejeda N. Vegetarian diets in Spain: temporal evolution through national health surveys and their association with healthy lifestyles. Endocrinol Diabetes Nutr. (2023) 70:1–8. doi: 10.1016/J.ENDIEN.2022.02.022, PMID: 37268353

[20] Li D. Effect of the vegetarian diet on non-communicable diseases. J Sci Food Agric. (2014) 94:169–73. doi: 10.1002/JSFA.6362 23965907

[21] Dakin BC Ching AE Teperman E Klebl C Moshel M Bastian B. Prescribing vegetarian or flexitarian diets leads to sustained reduction in meat intake. Appetite. (2021) 164:105285. doi: 10.1016/J.APPET.2021.105285, PMID: 33930494

[22] Sabaté J Soret S. Sustainability of plant-based diets: back to the future. Am J Clin Nutr. (2014) 100:476S–82S. doi: 10.3945/AJCN.113.071522, PMID: 24898222

[23] Mathur MB Robinson TN Reichling DB Gardner CD Nadler J Bain PA. Reducing meat consumption by appealing to animal welfare: protocol for a meta-analysis and theoretical review. Syst Rev. (2020) 9:3. doi: 10.1186/S13643-019-1264-5, PMID: 31907028 PMC6945605

[24] Satija A Hu FB. Plant-based diets and cardiovascular health. Trends Cardiovasc Med. (2018) 28:437–41. doi: 10.1016/J.TCM.2018.02.004, PMID: 29496410 PMC6089671

[25] Clarys P Deliens T Huybrechts I Deriemaeker P Vanaelst B De Keyzer W. Comparison of nutritional quality of the vegan, vegetarian, semi-vegetarian, pesco-vegetarian and omnivorous diet. Nutrients. (2014) 6:1318–32. doi: 10.3390/NU6031318, PMID: 24667136 PMC3967195

[26] Hobbs SH. Attitudes, practices, and beliefs of individuals consuming a raw foods diet. Explore (NY). (2005) 1:272–7. doi: 10.1016/J.EXPLORE.2005.04.015, PMID: 16781548

[27] Link LB Jacobson JS. Factors affecting adherence to a raw vegan diet. Complement Ther Clin Pract. (2008) 14:53–9. doi: 10.1016/J.CTCP.2006.12.005, PMID: 18243943 PMC3635096

[28] Hiney K Sypniewski L Rudra P Pezeshki A McFarlane D. Clinical health markers in dogs fed raw meat-based or commercial extruded kibble diets. J Anim Sci. (2021) 99:133. doi: 10.1093/JAS/SKAB133, PMID: 33939804 PMC8174467

[29] Ahmed F Cappai MG Morrone S Cavallo L Berlinguer F Dessì G. Raw meat based diet (RMBD) for household pets as potential door opener to parasitic load of domestic and urban environment. Revival of understated zoonotic hazards? A review. One Health. (2021) 13:100327. doi: 10.1016/J.ONEHLT.2021.100327, PMID: 34584928 PMC8455362

[30] Zhang Q Zhang C Wang H Ma Z Liu D Guan X. Intermittent fasting versus continuous calorie restriction: which is better for weight loss? Nutrients. (2022) 14:781. doi: 10.3390/NU14091781, PMID: 35565749 PMC9099935

[31] Maroofi M Nasrollahzadeh J. Effect of intermittent versus continuous calorie restriction on body weight and cardiometabolic risk markers in subjects with overweight or obesity and mild-to-moderate hypertriglyceridemia: a randomized trial. Lipids Health Dis. (2020) 19:216. doi: 10.1186/S12944-020-01399-0, PMID: 33028352 PMC7542333

[32] Schübel R Nattenmüller J Sookthai D Nonnenmacher T Graf ME Riedl L. Effects of intermittent and continuous calorie restriction on body weight and metabolism over 50 wk: a randomized controlled trial. Am J Clin Nutr. (2018) 108:933–45. doi: 10.1093/AJCN/NQY196, PMID: 30475957 PMC6915821

[33] Rynders CA Thomas EA Zaman A Pan Z Catenacci VA Melanson EL. Effectiveness of intermittent fasting and time-restricted feeding compared to continuous energy restriction for weight loss. Nutrients. (2019) 11:2442. doi: 10.3390/NU11102442, PMID: 31614992 PMC6836017

[34] Dong TA Sandesara PB Dhindsa DS Mehta A Arneson LC Dollar AL. Intermittent fasting: a heart healthy dietary pattern? Am J Med. (2020) 133:901–7. doi: 10.1016/J.AMJMED.2020.03.030, PMID: 32330491 PMC7415631

[35] North M Klas A Ling M Kothe E. A qualitative examination of the motivations behind vegan, vegetarian, and omnivore diets in an Australian population. Appetite. (2021) 167:105614. doi: 10.1016/J.APPET.2021.105614, PMID: 34329718

[36] Miki AJ Livingston KA Karlsen MC Folta SC McKeown NM. Using evidence mapping to examine motivations for following plant-based diets. Curr Dev Nutr. (2020) 4:nzaa013. doi: 10.1093/CDN/NZAA013, PMID: 32110769 PMC7042611

[37] World Medical Association Declaration of Helsinki. Ethical principles for medical research involving human subjects. Nurs Ethics. (2002) 9:105–9. doi: 10.1191/0969733002NE486XX 16010903

[38] Sandri E Piredda M De Maria M Mancin S Sguanci M Cabo A. Development and psychometric testing of the nutritional and social health habits scale (NutSo-HH): a methodological review of existing tools. MethodsX. (2024) 12:102768. doi: 10.1016/j.mex.2024.102768, PMID: 38883583 PMC11177200

[39] AI N Moncada RO. Quality of the Spanish diet according to the healthy eating index. Nutr Hosp. (2011) 26:330–6. doi: 10.1590/S0212-16112011000200014, PMID: 21666971

[40] Grupo Colaborativo de la Sociedad Española de Nutrición Comunitaria (SENC). Guías alimentarias para la población española; la nueva pirámide de la alimentación saludable. Nutr Hosp. (2016) 33:1–48. 10.20960/nh.827 28196425

[41] Jamovi. (2024). Open Statistical Software for the Desktop and Cloud. Available at: https://www.jamovi.org/ (Accessed April 15, 2024).

[42] Online Statistics Calculator. (2024). Hypothesis testing, t-test, chi-square, regression, correlation, analysis of variance, cluster analysis. Available at: https://datatab.net/ (Accessed April 15, 2024).

[43] Rodriguez Artalejo F Banegas JR Graciani MA Hernández Vecino R Rey Calero J. Food and nutrient consumption in Spain in the period 1940-1988. Analysis of its consistency with the Mediterranean diet. Med Clin. (1996) 106:161–8. 8684014

[44] Mandal S Simmons N Awan S Chamari K Ahmed I. Intermittent fasting: eating by the clock for health and exercise performance. BMJ Open Sport Exerc Med. (2022) 8:e001206. doi: 10.1136/BMJSEM-2021-001206, PMID: 35070352 PMC8744103

[45] Effects of Intermittent Fasting on Health. Aging, and disease. N Engl J Med. (2020) 382:298. doi: 10.1056/NEJMx190038 31940711

[46] Malinowski B Zalewska K Węsierska A Sokołowska MM Socha M Liczner G. Intermittent fasting in cardiovascular disorders-an overview. Nutrients. (2019) 11:673. doi: 10.3390/NU11030673, PMID: 30897855 PMC6471315

[47] Liu K Liu B Heilbronn LK. Intermittent fasting: what questions should we be asking? Physiol Behav. (2020) 218:112827. doi: 10.1016/J.PHYSBEH.2020.112827, PMID: 32014525

[48] Dokova KG Pancheva RZ Usheva NV Haralanova GA Nikolova SP Kostadinova TI. Nutrition transition in Europe: east-west dimensions in the last 30 years–a narrative review. Front Nutr. (2022) 9:919112. doi: 10.3389/FNUT.2022.919112 35873435 PMC9301044

[49] da Costa GG da Conceição NG da Silva PA Simões BFT. Worldwide dietary patterns and their association with socioeconomic data: an ecological exploratory study. Glob Health. (2022) 18:31. doi: 10.1186/S12992-022-00820-W, PMID: 35279165 PMC8917745

[50] Gherasim A Arhire LI Nițǎ O Popa AD Graur M Mihalache L. The relationship between lifestyle components and dietary patterns. Proc Nutr Soc. (2020) 79:311–23. doi: 10.1017/S0029665120006898, PMID: 32234085 PMC7663317

[51] Arija V Pérez C Serra-Majem L Aranceta J. Gastronomy and nutrition in catalonia. Nutr Hosp. (2019) 36:78–85. doi: 10.20960/NH.02699 31232596

[52] Trabazo RL Pérez C De L Castro Pérez X Solla P. Atlantic diet. Nutrition and gastronomy in galicia. Nutr Hosp. (2019) 36:7–13. doi: 10.20960/NH.02686 31232586

[53] Dixon KA Michelsen MK Carpenter CL. Modern diets and the health of our planet: an investigation into the environmental impacts of food choices. Nutrients. (2023) 15:692. doi: 10.3390/NU15030692, PMID: 36771398 PMC9919910

[54] Nelson ME Hamm MW Hu FB Abrams SA Griffin TS. Alignment of healthy dietary patterns and environmental sustainability: a systematic review. Adv Nutr. (2016) 7:1005–25. doi: 10.3945/AN.116.012567, PMID: 28140320 PMC5105037

[55] Jorcano MQ. Nutrition and gastronomy in the community of La Rioja. Evolution of the food model, gastronomic identity and nutritional evaluation of the diet. Nutr Hosp. (2019) 36:47–55. doi: 10.20960/NH.02692 31232594

[56] Jaraíz Arias JF Perea AG. Nutrition and gastronomy in Extremadura. Nutr Hosp. (2019) 36:102–4. doi: 10.20960/NH.02704, PMID: 31232600

[57] Jiménez Monreal AM Antonia Murcia Tomás M Martínez TM. Nutrition and gastronomy in the community of Murcia Region. Nutr Hosp. (2019) 36:92–7. doi: 10.20960/NH.02702 31232599

[58] Wozniak H Larpin C De Mestral C Guessous I Reny JL Stringhini S. Vegetarian, pescatarian and flexitarian diets: sociodemographic determinants and association with cardiovascular risk factors in a Swiss urban population. Br J Nutr. (2020) 124:844–52. doi: 10.1017/S0007114520001762, PMID: 32418548 PMC7525113

[59] Allès B Baudry J Méjean C Touvier M Péneau S Hercberg S. Comparison of sociodemographic and nutritional characteristics between self-reported vegetarians, vegans, and meat-eaters from the nutrinet-santé study. Nutrients. (2017) 9:1023. doi: 10.3390/NU9091023, PMID: 28926931 PMC5622783

[60] Wardle J Haase AM Steptoe A Nillapun M Jonwutiwes K Bellisle F. Gender differences in food choice: the contribution of health beliefs and dieting. Ann Behav Med. (2004) 27:107–16. doi: 10.1207/S15324796ABM2702_5, PMID: 15053018

[61] Wu B Goins RT Laditka JN Ignatenko V Goedereis E. Gender differences in views about cognitive health and healthy lifestyle behaviors among rural older adults. Gerontologist. (2009) 49:S72–8. doi: 10.1093/GERONT/GNP077, PMID: 19525219

[62] Kim H Caulfield LE Rebholz CM. Healthy plant-based diets are associated with lower risk of all-cause mortality in US adults. J Nutr. (2018) 148:624–31. doi: 10.1093/JN/NXY019, PMID: 29659968 PMC6669955

[63] Bidmon S Terlutter R. gender differences in searching for health information on the internet and the virtual patient-physician relationship in Germany: exploratory results on how men and women differ and why. J Med Internet Res. (2015) 17:e156. doi: 10.2196/JMIR.4127, PMID: 26099325 PMC4526954

[64] Alcorta A Porta A Tárrega A Alvarez MD Pilar VM. Foods for plant-based diets: challenges and innovations. Food Secur. (2021) 10:293. doi: 10.3390/FOODS10020293, PMID: 33535684 PMC7912826

[65] Chard E Bergstad CJ Steentjes K Poortinga W Demski C. Gender and cross-country differences in the determinants of sustainable diet intentions: a multigroup analysis of the UK, China, Sweden, and Brazil. Front Psychol. (2024) 15:969. doi: 10.3389/FPSYG.2024.1355969, PMID: 38487654 PMC10937452

[66] Shalabi H Hassan AS FA AL-Z Alarbeidi AH Mesawa M Rizk H. Intermittent Fasting: Benefits, Side Effects, Quality of Life, and Knowledge of the Saudi Population. Cureus. (2023) 15:e34722. doi: 10.7759/CUREUS.34722, PMID: 36909028 PMC9998115

[67] Alnasser A Almutairi M. Considering intermittent fasting among Saudis: insights into practices. BMC Public Health. (2022) 22:592. doi: 10.1186/S12889-022-12908-4, PMID: 35346130 PMC8959076

[68] Collier R. Intermittent fasting: the next big weight loss fad. CMAJ. (2013) 185:E321–2. doi: 10.1503/CMAJ.109-4437, PMID: 23529969 PMC3652955

[69] Conde-Pipó J Mora-Fernandez A Martinez-Bebia M Gimenez-Blasi N Lopez-Moro A Latorre JA. Intermittent fasting: does it affect sports performance? A systematic review. Nutrients. (2024) 16:168. doi: 10.3390/NU16010168, PMID: 38201996 PMC10780856

[70] de Azevedo FR Ikeoka D Caramelli B. Effects of intermittent fasting on metabolism in men. Rev Assoc Med Bras. (2013) 59:167–73. doi: 10.1016/J.RAMB.2012.09.003 23582559

[71] Silverii GA Cresci B Benvenuti F Santagiuliana F Rotella F Mannucci E. Effectiveness of intermittent fasting for weight loss in individuals with obesity: a meta-analysis of randomized controlled trials. Nutr Metab Cardiovasc Dis. (2023) 33:1481–9. doi: 10.1016/J.NUMECD.2023.05.005, PMID: 37248144

[72] Sanchez-Sabate R Sabaté J. Consumer attitudes towards environmental concerns of meat consumption: a systematic review. Int J Environ Res Public Health. (2019) 16:1220. doi: 10.3390/IJERPH16071220, PMID: 30959755 PMC6479556

[73] Siegrist M Hartmann C. Impact of sustainability perception on consumption of organic meat and meat substitutes. Appetite. (2019) 132:196–202. doi: 10.1016/J.APPET.2018.09.016, PMID: 30322657

[74] Cramer H Kessler CS Sundberg T Leach MJ Schumann D Adams J. Characteristics of Americans choosing vegetarian and vegan diets for health reasons. J Nutr Educ Behav. (2017) 49:561–567.e1. doi: 10.1016/J.JNEB.2017.04.011 28689610

[75] Bozzola E Spina G Agostiniani R Barni S Russo R Scarpato E. The use of social media in children and adolescents: scoping review on the potential risks. Int J Environ Res Public Health. (2022) 19:9960. doi: 10.3390/IJERPH19169960, PMID: 36011593 PMC9407706

[76] Bitto Urbanova L Holubcikova J Madarasova Geckova A Van Dijk JP Reijneveld SA. Adolescents exposed to discrimination: Are they more prone to excessive internet use? BMC Pediatr. (2020) 20:1–7. doi: 10.1186/S12887-020-02241-3/TABLES/2 32842980 PMC7448512

[77] Layland EK Hill BJ Nelson LJ. Freedom to explore the self: how emerging adults use leisure to develop identity. J Posit Psychol. (2018) 13:78–91. doi: 10.1080/17439760.2017.1374440, PMID: 29276528 PMC5739337

[78] Palmer AK Jensen MD. Metabolic changes in aging humans: current evidence and therapeutic strategies. J Clin Invest. (2022) 132:451. doi: 10.1172/JCI158451, PMID: 35968789 PMC9374375

[79] Song DK Kim YW. Beneficial effects of intermittent fasting: a narrative review. J Yeungnam Med Sci. (2023) 40:4–11. doi: 10.12701/JYMS.2022.00010, PMID: 35368155 PMC9946909

[80] Giezenaar C Chapman I Luscombe-Marsh N Feinle-Bisset C Horowitz M Soenen S. Ageing is associated with decreases in appetite and energy intake–a meta-analysis in healthy adults. Nutrients. (2016) 8:28. doi: 10.3390/NU8010028 26751475 PMC4728642

[81] Raghupathi V Raghupathi W. The influence of education on health: An empirical assessment of OECD countries for the period 1995-2015. Arch Public Health. (2020) 78:1–18. doi: 10.1186/S13690-020-00402-5/FIGURES/17 32280462 PMC7133023

[82] Hamulka J Wadolowska L Hoffmann M Kowalkowska J Gutkowska K. Effect of an education program on nutrition knowledge, attitudes toward nutrition, diet quality, lifestyle, and body composition in polish teenagers. The ABC of healthy eating project: design, protocol, and methodology. Nutrients. (2018) 10:1439. doi: 10.3390/NU10101439 30720795 PMC6213798

[83] Sandri E Pardo J Cantín Larumbe E Cerdá Olmedo G Falcó A. Analysis of the influence of educational level on the nutritional status and lifestyle habits of the young Spanish population. Front Public Health. (2024) 12:1341420. doi: 10.3389/FPUBH.2024.1341420, PMID: 38651128 PMC11033505

[84] Darmon N Drewnowski A. Does social class predict diet quality? Am J Clin Nutr. (2008) 87:1107–17. doi: 10.1093/AJCN/87.5.1107, PMID: 18469226

[85] Anderson E Wei R Liu B Plummer R Kelahan H Tamez M. Improving healthy food choices in low-income settings in the United States using behavioral economic-based adaptations to choice architecture. Front Nutr. (2021) 8:734991. doi: 10.3389/FNUT.2021.734991, PMID: 34692747 PMC8526839

[86] Sandri E Cantín Larumbe E Part-Ferrer R Ferrer-Torregrosa J Fernández-Ehrling N. Diet and lifestyle in the Spanish population and their relationship with sociodemographic variables: a descriptive study. Food Secur. (2023) 12:3409. doi: 10.3390/FOODS12183409, PMID: 37761118 PMC10527864

[87] Shepon A Eshel G Noor E Milo R. The opportunity cost of animal based diets exceeds all food losses. Proc Natl Acad Sci USA. (2018) 115:3804–9. doi: 10.1073/pnas.1713820115, PMID: 29581251 PMC5899434

[88] Melina V Craig W Levin S. Position of the academy of nutrition and dietetics: vegetarian diets. J Acad Nutr Diet. (2016) 116:1970–80. doi: 10.1016/J.JAND.2016.09.025, PMID: 27886704

[89] Han SN. Vegetarian diet for cardiovascular disease risk reduction: cons. J Lipid Atheroscler. (2023) 12:323–8. doi: 10.12997/JLA.2023.12.3.323, PMID: 37800105 PMC10548188

[90] Cerrillo I Saralegui-Díez P Morilla-Romero-de-la-Osa R González de Molina M Guzmán GI. Nutritional analysis of the Spanish population: a new approach using public data on consumption. Int J Environ Res Public Health. (2023) 20:1642. doi: 10.3390/IJERPH20021642, PMID: 36674397 PMC9867222

[91] Santos-Buelga C González-Manzano S González-Paramás AM. Wine, polyphenols, and Mediterranean diets. What else is there to say? Molecules. (2021) 26:5537. doi: 10.3390/MOLECULES26185537, PMID: 34577008 PMC8468969

[92] Rodríguez-Rodríguez E Aparicio A Sánchez-Rodríguez P Lorenzo-Mora AM López-Sobaler AM Ortega RM. Vitamin D deficiency in Spanish population. Importance of egg on nutritional improvement. Nutr Hosp. (2019) 36:3–7. doi: 10.20960/NH.02798, PMID: 31368328

[93] Ditano-Vázquez P Torres-Peña JD Galeano-Valle F Pérez-Caballero AI Demelo-Rodríguez P Lopez-Miranda J. The fluid aspect of the Mediterranean diet in the prevention and management of cardiovascular disease and diabetes: the role of polyphenol content in moderate consumption of wine and olive oil. Nutrients. (2019) 11:2833. doi: 10.3390/NU11112833, PMID: 31752333 PMC6893438

[94] Tran E Dale HF Jensen C Lied GA. Effects of plant-based diets on weight status: a systematic review. Diabetes Metab Syndr Obes. (2020) 13:3433–48. doi: 10.2147/DMSO.S272802, PMID: 33061504 PMC7533223

[95] WHO. Global recommendations on physical activity for health. Geneva: WHO Library Cataloguing-in-Publication (2010), 1–58.

[96] Hetherington MM. Eating disorders: diagnosis, etiology, and prevention. Nutrition. (2000) 16:547–51. doi: 10.1016/S0899-9007(00)00320-8 10906551

[97] Dillman DA Eltinge JL Groves RM Little RJA. Evaluating Nonresponse Error in Mail Surveys. New York, NY: Elsevier (2002). 197–212.

[98] Singer E Van Hoewyk J Maher MP. Experiments with incentives in telephone surveys. Public Opin Q. (2000) 64:171–88. doi: 10.1086/317761 10984332

